# Intrauterine Fetal Demise: A Rare Complication of Wernicke's Encephalopathy Secondary to Hyperemesis Gravidarum

**DOI:** 10.7759/cureus.47270

**Published:** 2023-10-18

**Authors:** Anthony Pham, Robin Okpara, Nancy Rollins, Roy Jacob

**Affiliations:** 1 Department of Radiology, Texas Tech University Health Sciences Center School of Medicine, Lubbock, USA; 2 Department of Radiology, Texas Tech University Health Sciences Center, Lubbock, USA

**Keywords:** wernicke encephalopathy, vitamin b1 deficiency, intrauterine fetal demise, nonalcoholic wernicke’s encephalopathy, nausea and vomiting in pregnancy

## Abstract

Wernicke's encephalopathy (WE) is an acute neurological disorder caused by severe thiamine deficiency that manifests with a common range of clinical features including a triad of global confusion state, ophthalmoplegia, and ataxia. Though frequently associated with the alcohol-dependent population, WE has been seen in other patients where it often goes undiagnosed presumably due to rarity and variable clinical indications. In this case report, we highlight the importance of WE being considered as a differential diagnosis of acute encephalopathy particularly in women who have experienced fetal demise in conjunction with signs of malnourishment from hyperemesis gravidarum.

## Introduction

Wernicke’s encephalopathy (WE) is a severe and reversible neurological disorder manifesting from a significant thiamine (vitamin B1) deficiency, which, if not appropriately treated, can progress to Korsakoff syndrome, a permanent neurologic disorder. The classic triad of encephalopathy, oculomotor dysfunction, and gait ataxia occurs in 16%-33% of patients at presentation [1]. Although originally thought to commonly be associated with chronic alcoholism, only 50% of WE cases are due to alcoholism [2]. In the absence of alcoholism, WE may go unrecognized and untreated in other patient populations, such as those with a history of hyperemesis gravidarum. Here in this report, we describe such a case.

This article was previously presented as a meeting abstract at the 2022 Texas American College of Physicians Annual Meeting on October 29.

## Case presentation

A previously healthy 25-year-old woman, gravida 1 para 1, presented to labour and delivery (L&D) triage services from a rural area with intrauterine fetal demise at week 16 of pregnancy complicated by hyperemesis gravidarum, refractory to ondansetron. A prior pregnancy was complicated by gestational hypertension and diabetes. The patient was noted to be drowsy with a persistently diminished level of consciousness, hypertension, and no focal neurologic deficits.

The patient was discharged in a good condition following delivery of the fetus and with episodes of nausea and vomiting having subsided. Subsequently, the patient presented to the ED 24 hours later with severely altered mental status and decreased responsiveness. The patient was intubated for airway protection and transferred to the ICU for further evaluation. On evaluation, the patient was sedated, afebrile, hypertensive, and tachycardic. Pupils were constricted but reactive.

After readmission to the ED, Psychiatry was consulted for possible psychiatric symptoms. On examination, the patient was intubated, and history was gathered from the family, revealing that the patient began having episodes of emesis five weeks ago. At that time, she was able to hold a conversation and had no confusion or drowsiness. The memory loss, confusion, and delirium episodes began two weeks before her spontaneous vaginal delivery of her stillbirth. According to the family, the patient had been endorsing mood changes during her recent pregnancy but never had suicidal or homicidal ideations, hallucinations, delusions, paranoia, or self-injurious behavior. The patient also had no history of psychiatric illness or alcohol and substance abuse.

**Figure 1:**
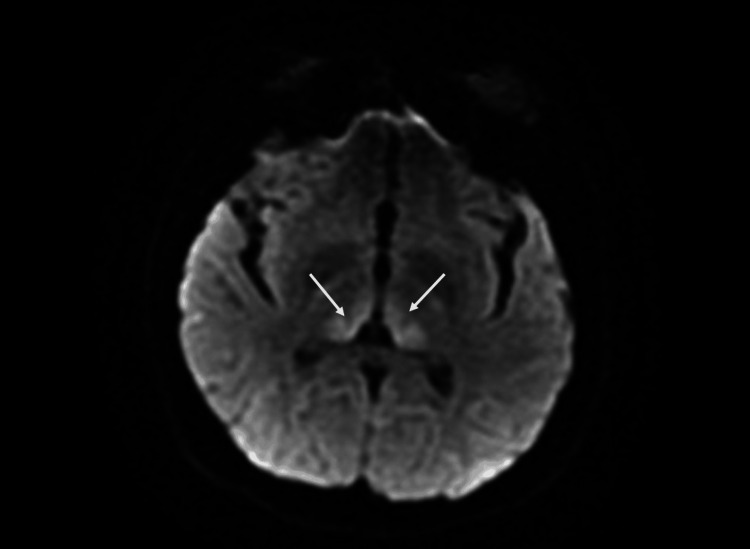
Axial diffusion-weighted image showing an abnormal signal in bilateral dorsal thalami

**Figure 2:**
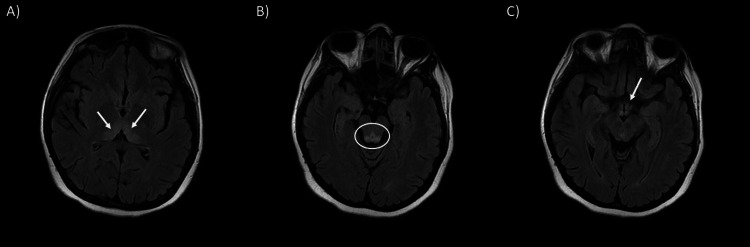
Axial T2 FLAIR images showing an increased signal in the (A) bilateral dorsal thalami, (B) periaqueductal white matter and (C) bilateral mammillary bodies FLAIR, fluid-attenuated inversion recovery

Head CT and CT angiography were normal. Brain MRI done on day 2 of ICU admission showed hyperintensities in the medial posterior thalamus, bilateral mammillary body, and periaqueductal region consistent with Wernicke’s encephalopathy but was otherwise normal (Figures 1, 2). High dose IV thiamine replacement therapy was immediately initiated with marked clinical improvements. The patient subsequently stabilized and self-extubated overnight. Neurological examination at the time showed equal and reactive bilateral pupils; extraocular movements were intact and no other neurological deficits were noted. The patient was alert and awake, but followed only a few commands.

Neurology was consulted for Wernicke's encephalopathy diagnosis. On evaluation, the patient was alert and oriented to person, time, place, and situation. The family noted some memory loss and confabulation. On physical examination, ophthalmoplegia was appreciated. Wernicke's encephalopathy was mostly likely secondary to thiamine deficiency due to her period of malnutrition during her severe history of hyperemesis gravidarum for five weeks.

Treatment

The patient received intravenous thiamine 500 mg replacement three times a day for five days, along with other vitamin supplements (B9 complex) and IV fluids. She then transitioned to oral thiamine supplementation until hospital discharge.

Outcome and follow-up

The patient was discharged home in a good condition after a total hospital stay of 10 days due to rehab and physical therapy. A subsequent follow-up after four months with Neurology showed total resolution of her confusion and amnesia as well as improvement in her horizontal nystagmus and gait ataxia. Repeat MRI done one year after the incident showed marked interval improvements and near-complete resolution of previously identified findings consistent with WE.

## Discussion

Wernicke’s encephalopathy is caused by thiamine deficiency, an essential water-soluble vitamin [3]. Thiamine pyrophosphate is predominantly found in the brain and is an essential cofactor for several vital enzymes, including transketolase, pyruvate dehydrogenase, and alpha-ketoglutarate, which are important for carbohydrate metabolism [4]. Thiamine deficiency results in neuronal apoptosis or necrosis [5]. The body stores an average of 25-30 mg of thiamine, sufficient for about 18 days, showing that a few weeks of dietary thiamine deficiency is enough to deplete the body's stores [5]. The recommended daily requirement is 0.4 mg/1000 kcal, increasing to 1.5 mg/day during pregnancy and lactation [6].

WE is most commonly seen in alcoholics, but can also occur in patients with malnutrition due to hyperemesis, malignancy, hemodialysis, starvation, and gastric surgery [7]. While rare, WE is a known complication of hyperemesis gravidarum, resulting from a combination of poor nutritional intake, frequent vomiting, and increased metabolic demands of pregnancy [8]. WE following hyperemesis gravidarum generally occurs at 14-16 weeks of gestation following two to three weeks of vomiting [9]. This was also the case for our patient, who presented at 16 weeks of gestation with a history of vomiting for five weeks.

Confusion is the most common presenting symptom, followed by ocular problems and staggering gait in WE [3]. However, in most cases, only one or two symptoms will be present. Therefore, diagnosis of WE should not solely rely on the presence of the classic triad without consideration of pregnancy and nutritional status [10]. WE should be suspected in a pregnant patient with a presentation of persistent vomiting and development of neurologic alteration, and intravenous thiamine replacement should urgently be started on an empiric basis [8].

While WE is reversible, serious complications can occur in pregnant women and unborn neonates. In approximately 80% of patients, untreated WE leads to Korsakoff syndrome with irreversible deficits in anterograde and retrograde memory, confabulation, and apathy [4]. Early pregnancy manifestations of WE can lead to preterm delivery, miscarriage, and intrauterine growth retardation [11].

Brain MRI is the imaging modality of choice in neurologic disorders, with a sensitivity of 53% and specificity of 93% in Wernicke’s encephalopathy [5]. Typical MRI findings include edema in the mammillary bodies, medial thalami, walls of the third ventricle, mesencephalon, brainstem, and superior vermis of the cerebellum and periaqueductal regions.

There are no universally accepted guidelines regarding the optimal dose, best route, and time of administration of thiamine in the setting of WE. The European Federation of Neurological Societies recommends that thiamine should be given 200 mg three times daily intravenously, started before any carbohydrate, and a regular diet should be initiated immediately after thiamine infusion [12]. Treatment should be continuously given until there is no further improvement in signs and symptoms of deficiency.

## Conclusions

Wernicke’s encephalopathy is a rare and life-threatening neurological disease complicating pregnancy in the setting of hyperemesis gravidarum with nonspecific signs and symptoms, or possibly in combination with other medical conditions. WE should be considered in any pregnant woman with severe hyperemesis, malnutrition, and vomiting, with complaints of neurological symptoms. Prompt treatment with thiamine can prevent both maternal and fetal morbidity.
